# Ready…Go: Amplitude of the fMRI Signal Encodes Expectation of Cue Arrival Time

**DOI:** 10.1371/journal.pbio.1000167

**Published:** 2009-08-04

**Authors:** Xu Cui, Chess Stetson, P. Read Montague, David M. Eagleman

**Affiliations:** 1 Program in Structural and Computational Biology and Molecular Biophysics, Baylor College of Medicine, Houston, Texas, United States of America; 2 Computation and Neural Systems Program, California Institute of Technology, Pasadena, California, United States of America; 3 Department of Neuroscience, Baylor College of Medicine, Houston, Texas, United States of America; 4 Menninger Department of Psychiatry and Behavioral Sciences, Baylor College of Medicine, Houston, Texas, United States of America; University of Minnesota, United States of America

## Abstract

What happens when the brain awaits a signal of uncertain arrival time, as when a sprinter waits for the starting pistol? And what happens just after the starting pistol fires? Using functional magnetic resonance imaging (fMRI), we have discovered a novel correlate of temporal expectations in several brain regions, most prominently in the supplementary motor area (SMA). Contrary to expectations, we found little fMRI activity during the waiting period; however, a large signal appears after the “go” signal, the amplitude of which reflects learned expectations about the distribution of possible waiting times. Specifically, the amplitude of the fMRI signal appears to encode a cumulative conditional probability, also known as the cumulative hazard function. The fMRI signal loses its dependence on waiting time in a “countdown” condition in which the arrival time of the go cue is known in advance, suggesting that the signal encodes temporal probabilities rather than simply elapsed time. The dependence of the signal on temporal expectation is present in “no-go” conditions, demonstrating that the effect is not a consequence of motor output. Finally, the encoding is not dependent on modality, operating in the same manner with auditory or visual signals. This finding extends our understanding of the relationship between temporal expectancy and measurable neural signals.

## Abstract 2

A neuroimaging study reveals novel insights into how the brain responds to an anticipated event, such as a starting gun or responding to a green light.

## Author Summary

Like the sprinter waiting for the starting pistol, all animals develop expectations about when events will occur in time. We explored the neural correlates of readiness and expectation using functional magnetic resonance imaging (fMRI), and found areas of the brain in which the fMRI signal remains at baseline during the waiting period and rises sharply after a cue to react (a “go” cue). Strikingly, the amplitude of the rise reflects a function of the probability of an event occurring at that time. The dependence on probability remains even in the absence of a motor act (that is, not pressing a button when the go cue appears). When the arrival time of the go cue is known in advance, the expectation-dependent signal disappears, indicating that this brain response reflects expectation, not simply elapsed time. These results match up with prior studies of expectation in the brain, with one important difference: previously, electrophysiology experiments showed that expectation is encoded by a build-up of spiking activity as the waiting period progresses, while our fMRI data reveal a signature of expectation that becomes apparent after the waiting concludes. We discuss the apparent mismatch between these different technologies for measuring expectation-related activity in the brain.

## Introduction

How long before the traffic light changes from red to green? When will the person on the other end of the line pick up the phone? Is the kettle about to whistle? To allow preparation, planning, and efficient allocation of resources in the face of uncertain timing, brains actively maintain expectations about the possible timing of future events [1]–[4]. Specifically, they extract temporal expectations when the arrival time of a stimulus is distributed with a probabilistic temporal structure.

The fact that brains learn temporal structure is exemplified by the finding that reaction time (RT) is modulated by changes in the temporal probability distribution between a warning signal (“ready”) and the imperative signal (“go”) [5]–[9]. For example, when the go signal is equally probable to appear at any one of a number of possible times, the RT is found to be faster for longer waiting periods. By the 1950s, the monotonically decreasing relationship of RT to the readiness period led to the hypothesis that RT depended on the a posteriori probability of the go cue [10] rather than the a priori probability; in other words, what matters is the probability that the cue will happen now *given that it has not already occurred*. The function describing this a posteriori probability distribution is called the hazard function [11]. The relation of RT to the hazard function has been verified by manipulating the probability distributions of the go-cue appearance times, and comparing the behavioral outcomes to the a posteriori functions [12]–[15].

The learning of temporal structure is also apparent in neural signals measured in single unit electrophysiology [14],[16]–[19] and EEG [20]–[22]. These studies have reported increasing neural signals that build over the course of the readiness period and typically resemble the hazard function. What remains unknown, however, is how learned temporal expectations relate to different signals in the brain, such as the blood oxygenation level dependent (BOLD) signal in functional magnetic resonance imaging (fMRI). Given the possible decoupling between action potentials and the fMRI signal [23],[24], it remains unknown whether neuroimaging would reveal a similar climbing activity or something quite different.

A less well-studied phase of the evolution of readiness-related movement is the transition between the readiness state and the baseline, post-“go” state. Given the relatively high level of electrical activity just before the go cue, one might expect large chemical changes to occur for the brain to resume its baseline state. These changes might be visible through a measurement technique such as fMRI. Several fMRI experiments have explored motor movements to temporally uncertain cues [7],[25],[26], but no event-related experiment has directly explored, to our knowledge, the pre- and post-go fMRI correlates of the temporal uncertainty itself, in which the only variable is the time of the go-cue.

We report here the results of such an experiment, in which participants in the fMRI scanner reacted as quickly as possible to a cue following a variable readiness period. Using this ready-go task, we looked for correlates of the readiness period in the BOLD response. Our experiment also allowed us to monitor the less well-studied transition between the time of the readiness period to the time of the baseline waiting.

As will be shown below, we found that activity in the supplementary motor area (SMA) and superior temporal gyrus (STG) was larger after longer periods of readiness. Strikingly, and contrary to the expectations from electrophysiology, we did not find any evidence of climbing activity in these areas. Rather, a large rise in the fMRI signal appeared immediately *after* the appearance of the go cue. The magnitude of the post-go signal was related to the probability of the wait time; we determined this by modulating the probability distribution in different trial blocks. We found the response to be well-fit by a cumulative hazard function, suggesting that the SMA and STG compute probabilistic expectations about waiting time. Electrophysiological evidence from monkey and human corroborate a role for the SMA in computing expectations about waiting time [16],[19],[20],[27],[28], although the form of the fMRI signal presented here differs in that it appears at the conclusion of the trial, as opposed to building up during the trial. Altogether, these results suggest a network of brain areas which construct temporal expectations in order to optimize reactions. These results further support the recent understanding that electrophysiological measures do not always yield a clear-cut prediction of the associated fMRI signals [24],[29].

## Results

### fMRI Signal Amplitude after the Go Cue Increases with Wait Time

**Figure 1:**
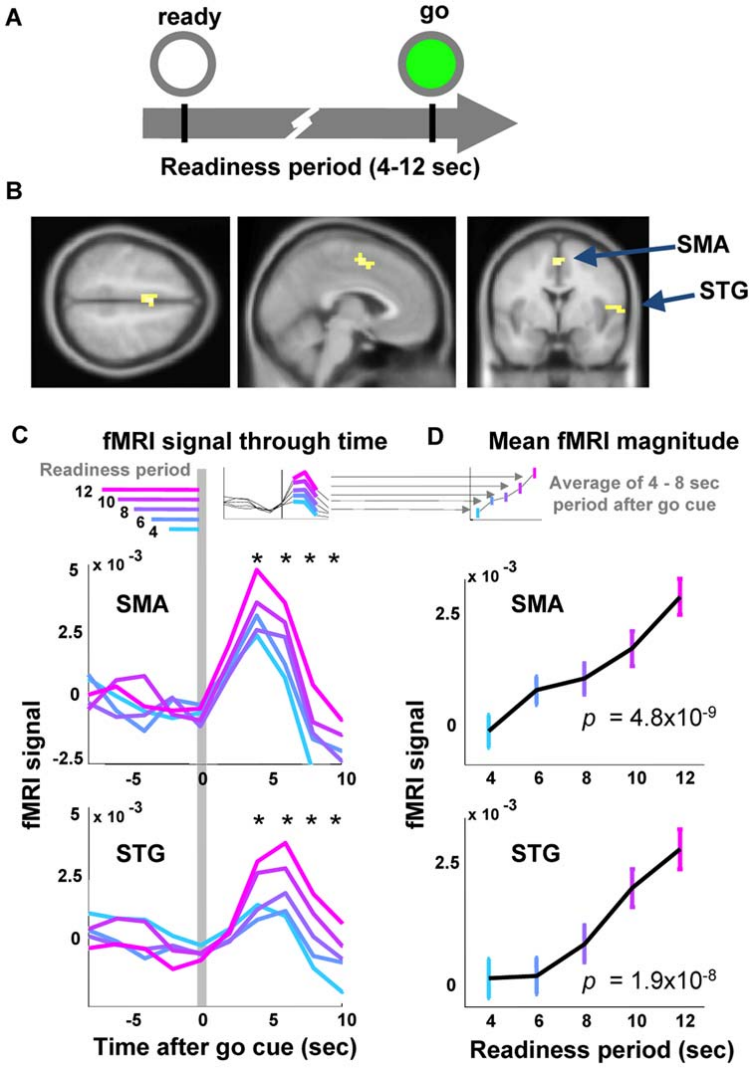
A longer readiness period correlates with a larger fMRI signal in SMA and STG. (A) In each trial, a gray ring (ready cue) appeared and remained on the screen. After several seconds (readiness period, 4–12 s, randomly interleaved), the ring became filled with green (go cue). Participants pressed a button as quickly as possible when they saw the green. (B) General linear model analysis (see Methods) identified the SMA (peak voxel coordinates in MNI space: −4, 0, 52) and STG (peak at 60, 9, −9) as regions whose activity was greater with increasing readiness periods (*p*<0.01, FDR corrected, cluster size >15). (C) Time series of the raw fMRI signal in the SMA and STG, averaged within different readiness periods. The vertical gray bar marks the arrival of the go cue. Each horizontal bar indicates a different readiness period; the left end of each bar represents the onset of the ready signal. The fMRI signal remained near baseline during the readiness period and rose sharply at the onset of the go signal. Longer readiness period caused higher blood flow after the appearance of the go cue. Asterisks represent the time points at which the fMRI signal amplitude correlates significantly with the time between the ready and go cue (*p*<0.001). Note that there is no significant correlation at the time points *before* the arrival of the go cue, only after. (D) The amplitude of the fMRI signal after the go cue correlates with the readiness period (*p*-values of the correlation, inset). Each point represents the average between measurements taken at 4 and 8 s *post*-go-cue (see inset). Error bars SEM.

Participants engaged in a reaction time experiment (Figure 1A). At the beginning of each trial, a gray ring (ready signal) appeared and remained on a black screen. After a readiness period of several seconds, the gray ring became filled with green (go signal). Participants were asked to press a button as quickly as possible when it became green. Readiness periods of 4, 6, 8, 10, or 12 s were randomized from trial to trial, according to a probability distribution (uniform, in this case), which remained constant throughout the block. Intertrial intervals were also randomized between 4 and 12 s.

Participants' reaction times were found to be a function of waiting time (Figure S1 and Text S1), a well known effect (known as variable foreperiod effect) that demonstrates the participants had learned the timing structure of the task [5]–[9].

To search for expectation-related activity in the fMRI signal, we designed a regressor to extract signals that were larger after longer waiting times, but was agnostic to the detailed timecourse of the signal (see Methods). At a threshold value of *p*<0.01 (false discovery rate [FDR] corrected for multiple comparisons), we found significant activity in two brain regions (Figure 1B): the SMA (peak at 0, 4, 52, in Montreal Neurological Institute [MNI] space), *t* = 5.63, ventral and slightly rostral to the midline, locating most voxels in the SMA but with some overlap in the preSMA [30] and [29] and STG (peak at 60, 9, −9, MNI, *t* = 5.63). The SMA has been previously implicated in time perception [21],[22],[28] and the timing of intention [31]–[33]. The STG has also been implicated in time and memory paradigms [28], although less prominently in the literature.

To understand the timecourse of the fMRI signal in these two regions, we plotted the activity in these two areas. We found that the signals in both the SMA and STG rose suddenly just *after* the appearance of the go signal (Figure 1C). (Due to hemodynamic delay, this rise presumably results from events occurring just around the time of the go cue.) More strikingly, the amplitude of this rise was highly significantly correlated with the readiness period, i.e., how long the participant had to wait (Figure 1D).

We found no evidence of expectation-related activity before the go-cue. This result surprised us, and we designed several other regressors that hypothesized the existence of signals that grew over the course of the waiting period (see Methods). The only significant climbing or sinking activity found in our task was in the visual cortices, and can be explained exclusively by the properties of our visual stimulus (see “No Evidence for Motor-Related Climbing Activity,” below). Therefore, we conclude that the expectation-related neural activity available to the fMRI technique appears largely after the conclusion of the waiting period.

### The Differential Post-Go Signal Does Not Depend on Motor Output

**Figure 2:**
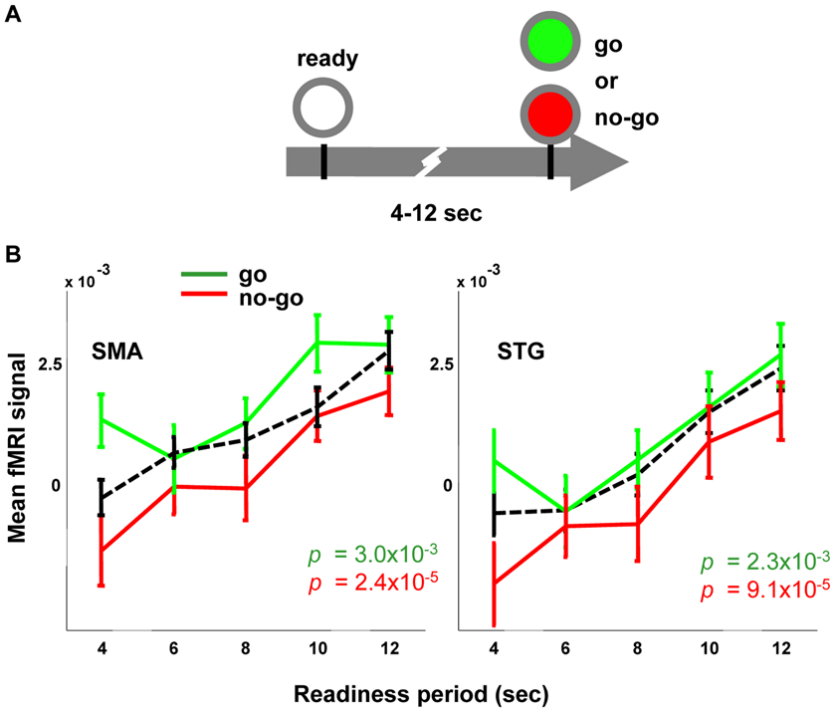
Longer readiness period causes higher fMRI signal in the absence of motor output. (A) The gray circle may be filled with red or green with equal probability. Participants are instructed not to press the button when they see red. (B) The post-trial fMRI signal amplitude is correlated with the readiness period in both the go (green) and no-go (red) trials (*p*-values of the correlation, inset). Note that baseline activity is slightly lower in the no-go condition, suggesting that motor output provides a constant signal offset that is independent of the waiting-time-dependent encoding described in this paper. For comparison, the dashed line shows the signal from the original experiment (Figure 1D). Error bars SEM.

While the significant differences in the fMRI signal between 8–12 s (Figure 1C and 1D) are not mirrored in the reaction time (Figure S1 and Text S1), it could nevertheless be possible that some aspect of the motor act played a role. To address whether the delayed fMRI signal is a consequence of motor output, we designed a second experiment (Figure 2A), which was almost identical to the first one except that the gray circle turns green (go signal) or red (no-go signal), each with probability 0.5. We found the same pattern of readiness period-dependent amplitude in both types of trials: a longer readiness period causes a larger post-go fMRI response (Figure 2B). Incorrect trials (trials in which participants pressed the button after a no-go signal, or in which they failed to press the button after a go signal) are only ∼3% of the total number of trials and they are not included in the analysis. Response inhibition is known to activate the SMA, which may contribute to the SMA activation in the no-go trials. However, there is no reason to expect response inhibition to show differential activity for the readiness period, which is the novel result in this case. The go/no-go results demonstrate that the differential fMRI amplitude is not simply a consequence of motor output.

### The Differential Post-Go Signal Disappears in the Absence of Uncertainty

**Figure 3:**
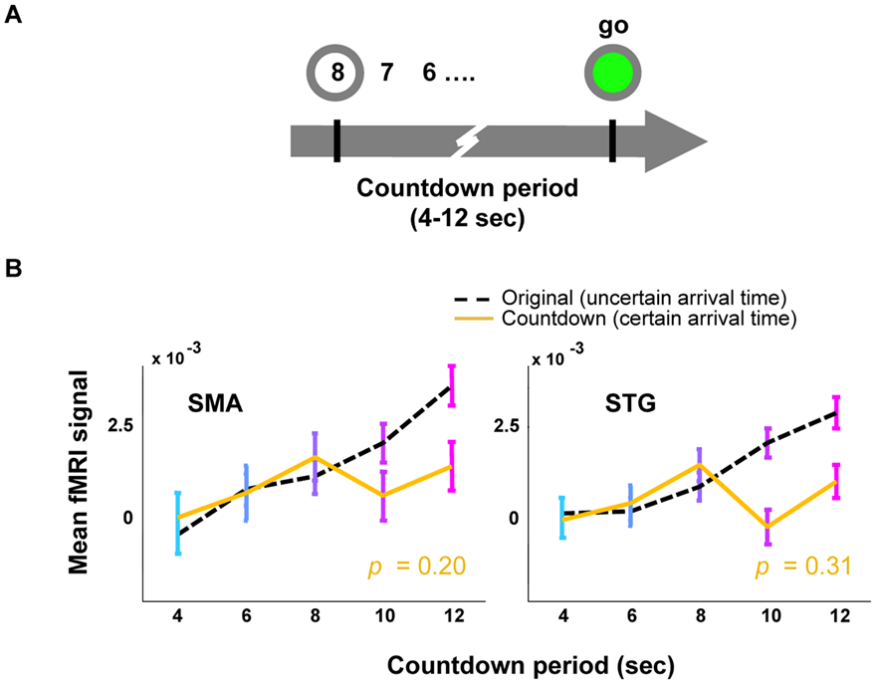
The post-go fMRI signal no longer correlates with the waiting period when there is certainty about the arrival time of the go cue. (A) In the countdown condition, a number in the middle of the gray ring counted down the seconds until the appearance of the go signal. (B) When there is no uncertainty about the arrival time of the go signal, the fMRI signal is independent of the readiness period (solid line, *p*-values of the correlation, inset). For comparison, the dashed line represents the fMRI signal in the original experiment (Figure 1), in which there is no countdown. Error bars SEM.

The post-go signal is modulated by the duration of the waiting period, and persists in the absence of a motor act. This suggests that it is a function of either expectancy or the duration of the waiting period itself. If the signal depends on expectancy, then it should lose its dependence on the duration of the readiness period in the absence of uncertainty. To test this prediction, we conducted an experiment in which a numeric display counted down the remaining seconds of the readiness period; the go signal occurred when the countdown reached zero (Figure 3A). As in the previous experiments, five possible readiness periods were randomly interleaved. In the absence of uncertainty about the arrival time of the go signal, the amplitude of the fMRI signal no longer correlated with the readiness period (Figure 3B). This is consistent with previous observations that heart rate increasingly slows during a waiting paradigm, but does not slow if the readiness period is counted down, i.e., there is no uncertainty about arrival time [34].

### Expectancy Modulates the Amplitude of the Post-Go Signal

**Figure 4:**
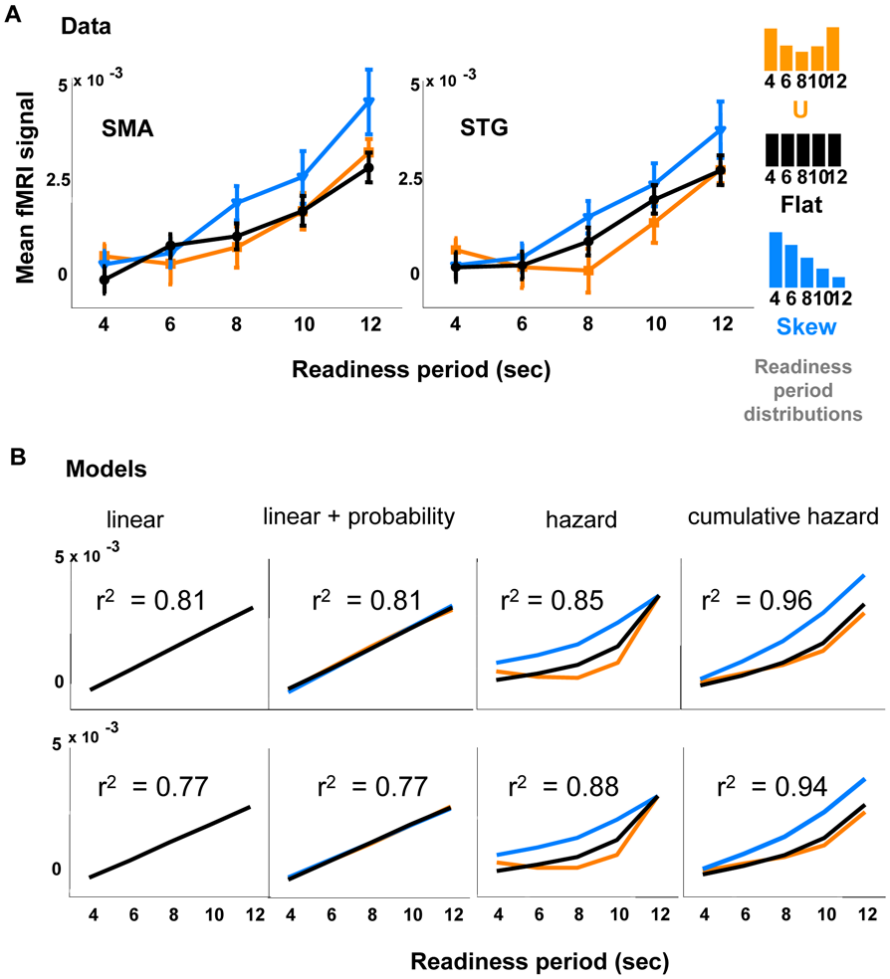
The fMRI signal is modulated by the probability distribution of the timing of go signal. (A) Inset shows three readiness period probability distributions for the SMA, as presented in different trial blocks. Black, orange, and blue lines represent flat, U-shaped, and skewed distributions of readiness period, respectively. In the flat distribution, the probability of each readiness period is 0.2. For the U-shaped block the probabilities are: 4 s, 0.28; 6 s, 0.16; 8 s, 0.12; 10 s, 0.16; 12 s, 0.28. For the skewed block: 4 s, 0.36; 6 s, 0.28; 8 s, 0.2; 10 s, 0.12; 12 s, 0.04. (B) Best fits for the four models described in the text. The cumulative hazard function provides the most accurate fit of the data. Specifically, by taking the residual differences between data and fit, and calculating a *t*-test between the cumulative hazard model and each of the others, we found in the SMA that the cumulative hazard fits significantly better than the other three (*p*<0.01, 0.01, 0.02, respectively). Another method, which directly tests the null hypothesis that the observed *r* ^2^ = 0.96 in our sample for the cumulative hazard model is from a population correlation *r* ^2^ = 0.85 (see Methods for full details), yields p<0.01, which is comparable to the previous method. In the STG, we find *p*<0.11 (*t*-test on residual) and *p*<0.15 (correlation coefficient distribution) between the cumulative hazard model and hazard model, indicating a trend favoring the cumulative hazard model.

We have shown that the signal appearing after the go cue is modulated by the expectancy of an uncertain cue, since it disappears in the absence of uncertainty. This predicts that the probability distribution of the timing of the go cue will have an effect on the post-go signal. To determine whether the temporal probability distribution influences the fMRI amplitude, or whether it is instead based solely on the total time waited, we conducted another experiment in which we manipulated the probability distribution of the readiness period (Figure 4). Three different distributions gave rise to different patterns of fMRI amplitudes (Figure 4A), suggesting a relationship between expectancy and the blood flow response. To elucidate this relationship, we proposed and tested four models (Figure 4B): (1) The fMRI amplitude depends only on the length of the readiness period, not on the probability distribution; (2) The amplitude depends on a linear combination of readiness period and probability; (3) The amplitude depends on the conditional probability (or hazard function), i.e., the probability that the go signal occurs at time *t* given it has not yet happened by *t*; (4) The activity depends on the cumulative conditional probability, i.e., the integral of the hazard function from time 0 to *t*.

The results of the modeling are shown in Figure 4B. As seen by the *r* ^2^ values, the cumulative hazard model appears to best explain the data. We note, however, that the hazard function by itself provides a qualitatively good fit as well, capturing some features of the data better than the cumulative hazard. It may be that the true signal is some combination of the two, representing a kind of leaky or forgetful accumulation. Further experiments will be required to address this possibility.

### Expectation Dependent Activity Generalizes to Auditory Cues

**Figure 5:**
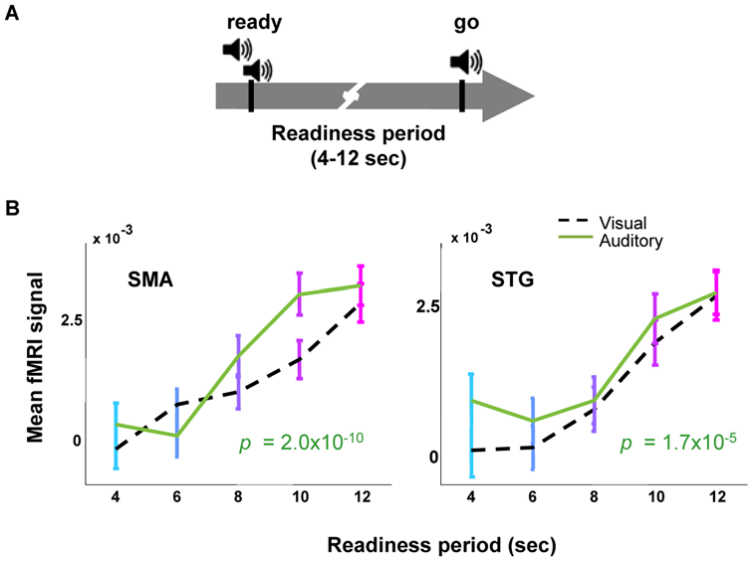
Evidence for an expectation-dependent fMRI signal generalizes to an auditory ready-go experiment. (A) Participants viewed a blank screen. A brief double beep served as the ready signal, and a brief single beep served as the go signal. (B) As in the visual condition (Figure 1), longer readiness periods correlate with higher post-go fMRI signal (*p*-values of the correlation inset). Solid line, data from the auditory experiment. For comparison, the dashed line shows the fMRI results from the visual experiment (Figure 1). Error bars SEM.

To determine if the effect we have described depends on the sensory modality, we repeated our original experiment with auditory cues. Here, a brief double beep was the ready signal, and a brief single beep was the go signal. The fMRI signal in both regions was almost identical in the auditory and visual conditions (Figure 5). This indicates that the readiness-period-dependent activity is not reliant on visual cues, but is a function of expectation more generally.

### No Evidence for Motor-Related Climbing Activity in the fMRI Signal

**Figure 6:**
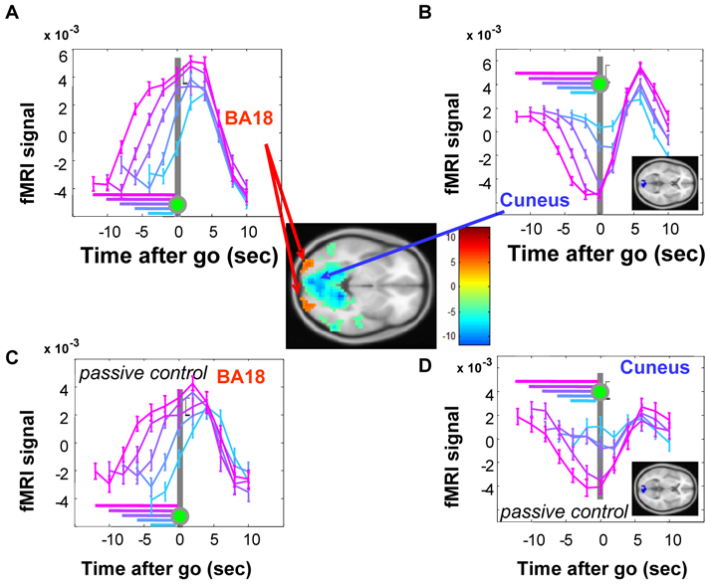
Climbing and sinking fMRI signals during the readiness period. Center inset: BA18 (peak coordinates: 28 −92 −8) are the only regions that show a climbing signal during the readiness period at *p*<0.001. More broadly distributed areas, most significantly in cuneus (peak coordinates: −8 −92 0) show a sinking signal at *p*<0.001. Color bar indicates *t*-value with degree of freedom 19. (A) Time series of the fMRI signal in BA18 around the go signal. (B) fMRI signal in cuneus (inset) showing sinking activity. This region is a subset of the regions shown in the center inset with a more stringent threshold of *p*<10^−8^. The dynamics of the sinking signal appear to mirror the climbing signal, suggesting the possibility that the sinking signal results from resource sharing (draining) with nearby climbing areas; however, the differential sizes of these regions (center inset) calls such a hypothesis into question. (C) fMRI signal in BA18 in the passive control experiment in which the visual stimuli are identical to the first experiment (Figure 1A) but no button press is required. Here a similar climbing signal is evident, indicating that the climbing activity in BA18 is not due to motor preparation. (D) fMRI signal in the cuneus in the passive control condition, showing that the sinking signal, like the climbing signal, is independent of motor preparation. Error bars SEM.

The pattern of activation we have reported was unexpected, given that previous electrophysiological [35]–[39] and fMRI studies [25] have reported climbing activity during the readiness period. We thus set out to understand where climbing activity could be found in our task. In a brain-wide search for climbing activity (see Methods), only bilateral Brodmann Area 18 (BA18, Figure 6) revealed a climbing fMRI signal during the readiness period (Figure 6A).

To determine whether the climbing activity in BA18 (Figure 6A) relates to motor preparation, we conducted a passive control experiment, identical to the first experiment except that participants did not press a button at the go signal. We found the same pattern of climbing activity in these regions in the passive control condition (Figure 6C), indicating that the climbing activity BA18 is not due to motor preparation. This conclusion is further supported by the lack of climbing activity in BA18 in the auditory condition (unpublished data). Similarly, we found a broadly distributed “sinking” fMRI signal, which was especially significant in the cuneus (Figure 6B); its presence in the passive control condition revealed that it did not depend on the motor preparation (Figure 6D). In summary, we did not find evidence for climbing or sinking activity associated with motor preparation in our experiment.

## Discussion

We have reported a BOLD signature of the readiness period (Figure 1). As measured by fMRI, activity in SMA and STG rises to a level that reflects the timing expectations from the previous waiting period. Our results are consistent with electrophysiological findings in monkey [14],[17]–[19], and human [21],[22], which show expectancy of the cue reflected in the brain activity. However, they differ in one important respect: the electrophysiology finds expectancy-related activity building during the readiness period [16],[20],[40]–[42], whereas our data reflect a delayed and accumulated signature of the expectancy. While other fMRI studies have shown uncertainty-related signals in motor areas (e.g. temporal and spatial uncertainty-related activity in premotor cortex and pre-SMA using a block design [26] and forebrain areas such as the middle frontal gyrus and cingulate [7]), the results shown here are the first, to our knowledge, from a simple reaction-time task to a single target, using an event-related design, which can reveal the exact temporal profile of the expectancy signal. Because the fMRI signal reported here appears at the end of the readiness period, our data support the possibility that it represents a final integration of the hazard-like signals that have been measured electrophysiologically in early visual areas [17], lateral intraparietal cortex [14],[18], or the SMA itself [19].

It is particularly telling that our results are found in the SMA and STG. The SMA is known to be involved in time estimation, as are nearby parts of the anterior cingulate cortex [2]. The STG has also been implicated in time and memory paradigms [28]. However, in previous studies the dorsolateral prefrontal cortex (DLPFC) has been implicated in foreperiod time estimation, as evidenced by the reduction of foreperiod-related reaction time increase in patients with lesions present in that location [8]. In one recent study by Coull et al. [28], the STG, SMA, and DLPFC were coactivated during a time estimation task, but only SMA and STG were significantly activated during the retrieval process. It is possible that the lack of DLPFC activation in our study means that SMA and STG are activated after the conclusion of an event requiring temporal estimation, while the DLPFC is only activated during the estimation process.

Of what potential use is such a delayed reflection of expectancy to the brain? It has been suggested to us that it may act as a prediction error signal, adjusting temporal expectations each time the brain experiences a new temporal event—and adjusting even more when the event is extremely improbable. This theory is appealing on the surface, particularly if you look at the response to the skew distribution (Figure 4), which is highest when the probability is low. This hypothesis awaits further theory and experiment. In the meantime, we more cautiously propose only that the activation represents a metabolic product, or by-product, of accumulated expectation-related activity.

Why have these effects only become apparent with the fMRI technique? Electrophysiology is highly biased toward recording from large excitatory pyramidal cells, and it may be that while these cells' activity climbs throughout the readiness period, inhibitory interneuronal activity concomitantly drops. Note that attentional modulation in V4 is found in two types of neurons: “out” cells, whose firing rate decreases during expectancy, and “in” cells, whose firing rates increase [17]. Assuming that the fMRI signal correlates with firing rate, declining and increasing firing rates in neighboring cells could theoretically counterbalance each other, resulting in a flattened fMRI response.

Another possible source of the difference between our findings and previous reports lies in the details of the tasks. Experiments that have explored the readiness period with electrophysiology [14],[19] and fMRI [25],[26] have used tasks involving movements to or attention toward multiple locations in visual space. In contrast, the task we describe here is simpler, involving only a fingerpress to a nonvisible button. It could be that attending to or planning more complicated movements toward multiple targets in visual space recruits circuitry that exhibits climbing activity, but which is not needed for a simple fast-reaction.

A recent fMRI study by Curtis and Connolly (2008) shows evidence of climbing activity in saccade-related areas [25]. While it is hard to reconcile the differences between our two reports, they may relate to the fact that we used keypresses instead of saccades, that our task structure was simpler (target location never varied), and that our delay range was twice the size of theirs. Interestingly, when we examine their data carefully, it appears that their plots from the transverse parietal sulcus show evidence of a post-go, duration-dependent amplitude similar to ours (Figure S2 and Text S1).

A final possible source of the difference between our post-cue fMRI signal and the building signals from the electrophysiology literature is the difference in measurement modalities. As yet, there is no consensus on the relationship of the fMRI signal to electrical activity in the brain [23]. We could entertain the speculative possibility that the metabolic signal measured by fMRI may not always be directly coupled to the information-carrying signal measured electrically. As the brain maintains a state of readiness, electrical activity uses resources. So as not to disrupt the delicate balance in activity required to maintain vigilance, the brain may “pay back” the energy debt with oxygenated blood flow only after the readiness period has ended. Further experiments will be required to determine whether it is possible to disconnect the signaling cascade between energy consumption and increased blood oxygenation. Like other recent demonstrations [24],[43],[44], our data may show that fMRI signal and single unit spiking can be decoupled. If the hypothesis that fMRI-measured metabolic activity could be decoupled from electrically measured spiking activity proved true, it would have far-reaching implications. By comparing spiking data to fMRI results on the same tasks, we could begin to get an idea of how the brain balances metabolic and computational needs on an energy budget. Given the numerous factors that comprise the fMRI signal [45],[46], future experiments with diverse technologies will be necessary to determine the physiological basis of the effect we report here.

## Methods

### Participants and Task Description

20 participants (11 male, average age 27.7 y) participated in the main experiment, the go/no-go experiment, and the U-distribution experiment; 21 participants (ten male, average age 27.5 y) participated in the skewed-distribution experiment, the countdown experiment, and the auditory experiment. 15 participants (seven male, average age 28.3 y) participated in the passive control experiment.

Each experiment consisted of 50 trials. For experiments other than the passive control, if participants pressed the button too slowly (reaction time longer than 600 ms), or if they pressed the button before the color changed to green, or if they pressed a button when the color turned red, they would see an error message. Participants were told they would be paid as a function of the number of errors they made. Erroneous trials were removed prior to further analysis. To balance hand usage, half of the participants used their right thumbs to press the button and the other half used their left thumbs in each experiment except the passive control.

### fMRI Methods

High-resolution T1-weighted scans were acquired using an MPRage sequence in a 3-Tesla scanner (Siemens). Functional run details: echo-planar imaging, gradient recalled echo; repetition time (TR) = 2,000 ms; echo time (TE) = 40 ms; flip angle = 90°; 64×64 matrix, 29 4-mm axial slices, yielding functional 3.4 mm×3.4 mm×4.0 mm voxels. Data analysis was performed using software package SPM2 (http://www.fil.ion.ucl.ac.uk/spm/software/spm2) and visualized using xjView (http://www.alivelearn.net/xjview/). Motion correction to the first functional scan was performed using a six-parameter rigid-body transformation [47]. The average of the motion-corrected images was coregistered to each individual's structural MRI using a 12-parameter affine transformation. The images were spatially normalized to the MNI template by applying a 12-parameter affine transformation, followed by a nonlinear warping using basis functions [47]. Images were then smoothed with an 8-mm isotropic Gaussian kernel and highpass filtered in the temporal domain (filter width of 128 s [48]).

We performed a general linear model regression on the data. Two regressors were delta functions occurring at the time of the go cue. One of these regressors corresponded to short wait periods (hrfs convolved with delta functions timed on go cues following readiness periods of <8 s), and the other corresponded to long wait periods (>8 s). A third regressor was added—an hrf convolved with delta functions timed on the ready cue—to account for the variance in the data created by that stimulus. A paired *t*-test was performed between beta values from the long and short readiness period regressors. Regions that survived the threshold (*p*<0.01, FDR corrected for multiple comparisons [49], cluster size >15 voxels) were subjected to further region of interest (ROI) analysis.

To search for climbing activity (Figure 6), we used several methods. (1) First, note that our original two regressors (described above) were designed to pull out climbing activity. Indeed, this method did pull out some of the activations shown on Figure 6, but in the opposite direction from Figure 1, and at much lower significance values. (2) Consistent with the reasoning from our original regressor, if a voxel displayed climbing activity *during* the readiness period, then the fMRI signal at the *end* of the readiness period (i.e., at the go cue) will be larger after longer readiness periods. However, unlike our original analysis, a more intuitive notion of climbing activity in the fMRI is that it should peak at the time of the go cue, not afterward. To search for voxels that satisfied this condition, we compared the amplitude exactly at the time of the go cue, *without* convolving with the hrf. This timing ensured that we were analyzing the result of activity during the delay period, rather than afterward. For each voxel, we chose the fMRI signals at the timing of go cue and performed a linear regression on the previous readiness period. We then performed a *t*-test on the beta values across participants. This analysis remains agnostic to the temporal pattern of the climbing activity, and only concentrates on where the activity ends up, just before the go cue appears. The result of this analysis is shown in Figure 5. The time series indicate that this method successfully pulls out activity that is greater for longer waiting periods. (3) Similar to Method 1, except that we performed a contrast between the BOLD amplitudes at the time of go for long waiting (10 and 12 s) and for short waiting (4 and 6 s). Whereas Method 1 hypothesizes a linear relationship between wait time and the height to which the activity might climb, this analysis remains agnostic to the exact functional form. Method 2 is the same as our main regressor, but without convolving with the hrf. The voxels produced by this latter analysis were qualitatively similar to those shown in Figure 6: BA18 and cuneus, but no significant activity (*p*<.001) anywhere else, including in the lateral intraparietal sulcus. (4) We also performed GLM analyses using either box-car or triangle regressors subtending the width of the readiness period. These methods successfully pulled out the areas that showed climbing activity as in Figure 6. These methods also revealed some other areas, such as several nuclei in the thalamus and basal ganglia, and the SMA and STG. Subsequent ROI analysis on these other areas showed that there was no climbing activity, although there was significant readiness-related activity appearing after the go-cue. Box-car or triangle approaches are not efficient at selectively revealing climbing activity because they will identify any regions whose activity is higher during the readiness period than during the intertrial interval. Time course analysis of the identified areas showed only transient visual responses to the ready cue, rather than climbing activity.

In the ROI analysis, the raw fMRI signal was extracted from each voxel in the region. Then the signal was averaged across voxels. The baseline was determined by a moving average with a window of +/−50 data points (i.e., +/−100 s). The baseline-subtracted signal was used for all region-of-interest time-course plots, and labeled according to percent change from the moving baseline. The signal amplitude was defined as the average of the signal at 4 s and 6 s data point after the go (or no-go) signal.

### Fitting Models to the fMRI Data

To determine the relationship between the temporal probability distribution of the go signal and the fMRI amplitude, we used linear regression to fit four models to the fMRI data (Figure 4B). In the equations below, *y* = the mean BOLD signal amplitude; *t* = time between ready and go signals (readiness period); *P(t)* = probability of go signal arriving at time *t*; and β is a fitting coefficient. Model 1: The fMRI amplitude depends only on the length of the readiness period, not on the probability distribution: *y* = β_1_ *t* + β_0_. Model 2: The amplitude depends on a linear combination of readiness period and probability: *y* = β_1_ *t* + β_2_ *P*(*t*) + β_0_. Weights were determined by fitting. Model 3: The amplitude depends on the conditional probability (or hazard function), i.e., the probability that the go signal occurs at time *t* given it has not yet happened by *t*: 
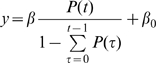
. Model 4: The activity depends on the cumulative conditional probability, i.e., the sum of the hazard function from time 0 (beginning of each trial) to *t* (the time “right now”):
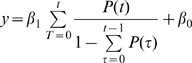



### Testing Significance between the Fitting of Hazard Model and Cumulative Hazard Model

We used two methods to investigate whether the cumulative hazard model fits the data significantly better than the hazard model: (1) *t*-test on residuals. After fitting the models, we calculated the residual for each model on each data point. Then for each data point, we calculated the difference of the absolute values of the residuals from the hazard model and the cumulative hazard model. We then performed a one sample *t*-test on these residual differences. (2) Distribution of correlation coefficient. We calculated the probability (*p*-value) that the observed sample correlation coefficient (*r* ^2^ = 0.96 from cumulative hazard model) is from a population correlation coefficient (*r* ^2^ = 0.85, the value we found for hazard model). The distribution of sample correlation coefficient *r*, given population correlation coefficient ρ and sample size *N*, is formulated as follows [50]:
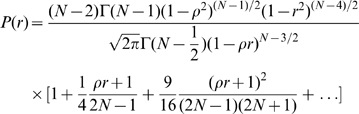
where Г is gamma function. We then calculated the area under this distribution curve where *r* ^2^>0.96, which is the *p*-value. Similar results were found with both methods; please see Figure 4B.

## Supporting Information

Figure S1 **Reaction times correlate with readiness periods on the basis of the underlying probability distributions (insets).** Circle area is proportional to the sample size within block. These data match the previously documented relationship between readiness period and reaction time (the variable foreperiod effect^1–3^), verifying that our participants learned the structure of the temporal probability distributions. Symbol diameter is proportional to the number of samples within each experiment. Error bars are standard error of the mean (SEM).
(0.17 MB TIF)

Figure S2 **A revision of the plot from Curtis and Connolly** [**24**] **that aligns the onset time of the go-cue reveals a result like that seen in our** **Figure 1** **.**
(0.91 MB TIF)

Text S1 **Supplementary material.**
(0.06 MB DOC)

## Abbreviations

BOLD: blood oxygenation level dependent
BA18: Brodmann Area 18
DLPFC: dorsolateral prefrontal cortex
FDR: false discovery rate
fMRI: functional magnetic resonance imaging
MNI: Montreal Neurological Institute
ROI: region of interest
RT: reaction time
SEM: standard error of the mean
SMA: supplementary motor area
STG: superior temporal gyrus
