# Supplementary figures and images for: Ready…Go: Amplitude of the fMRI Signal Encodes Expectation of Cue Arrival Time
Source: PLoS Biol. 2009 Aug 4;7(8):e1000167. doi: 10.1371/journal.pbio.1000167 (PMC2711330; doi:10.1371/journal.pbio.1000167)

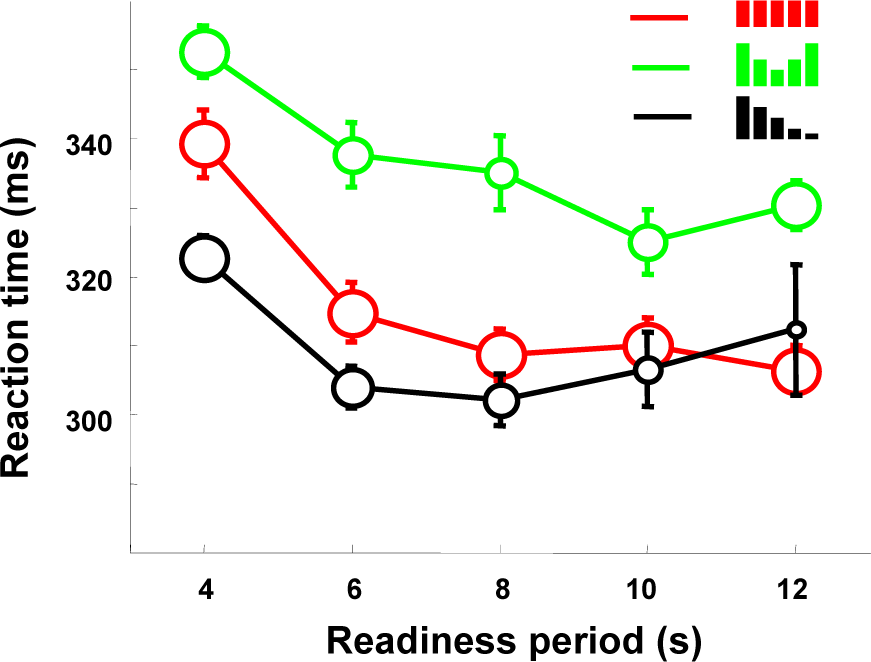

Supplement: Figure S1 — Reaction times correlate with readiness periods on the basis of the underlying probability distributions (insets). Circle area is proportional to the sample size within block. These data match the previously documented relationship between readiness period and reaction time (the variable foreperiod effect1–3), verifying that our participants learned the structure of the temporal probability distributions. Symbol diameter is proportional to the number of samples within each experiment. Error bars are standard error of the mean (SEM). (0.17 MB TIF) [file pbio.1000167.s001.tif]

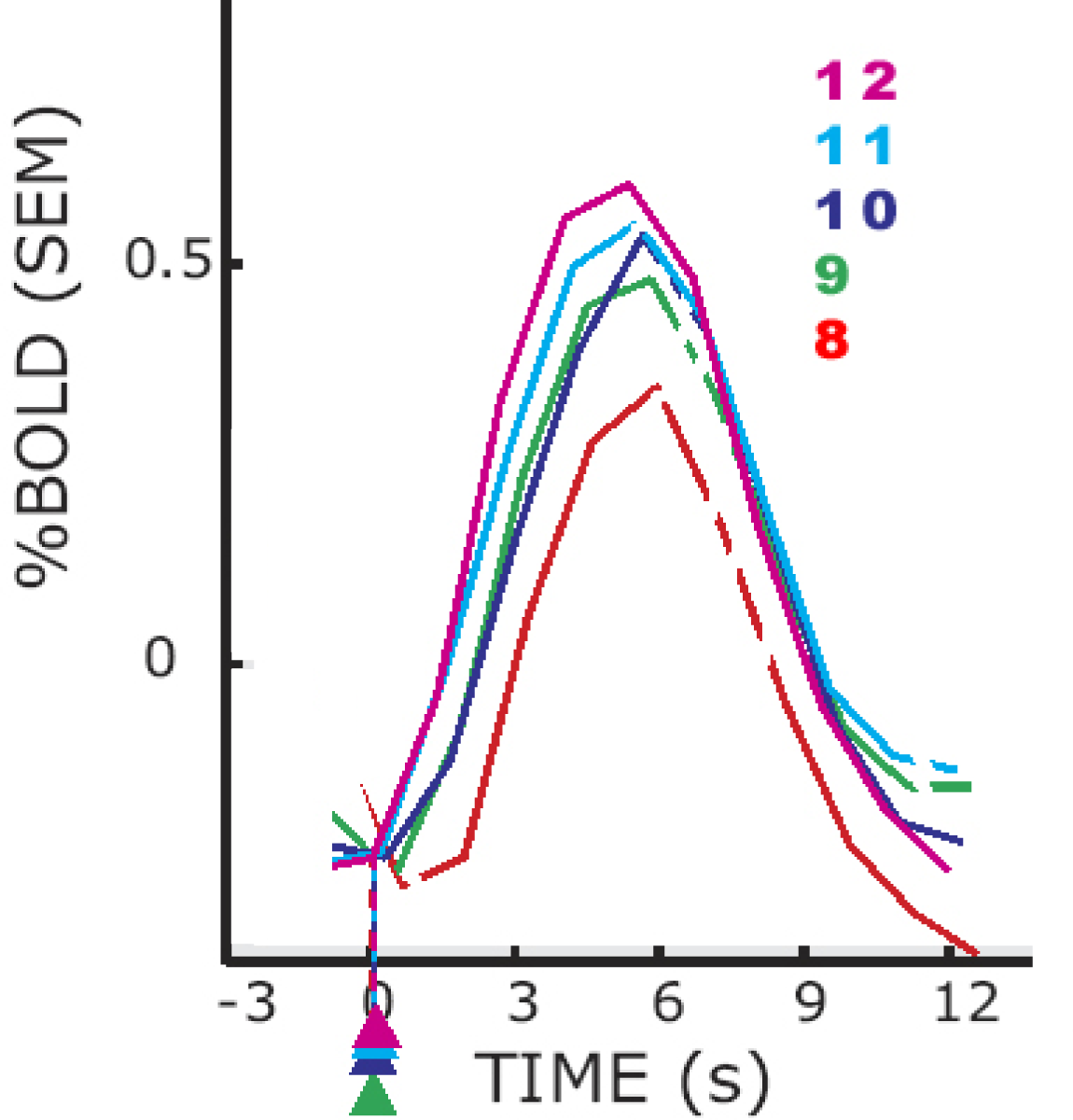

Supplement: Figure S2 — A revision of the plot from Curtis and Connolly [24] that aligns the onset time of the go-cue reveals a result like that seen in our Figure 1 . (0.91 MB TIF) [file pbio.1000167.s002.tif]
