# Supplementary material for: Ready…Go: Amplitude of the fMRI Signal Encodes Expectation of Cue Arrival Time
Source: PLoS Biol. 2009 Aug 4;7(8):e1000167. doi: 10.1371/journal.pbio.1000167 (PMC2711330; doi:10.1371/journal.pbio.1000167)
Supplement: Text S1 — Supplementary material. (0.06 MB DOC) [file pbio.1000167.s003.doc]

**Supplementary Material:**

**Ready…Go: Amplitude of the fMRI signal encodes expectation of cue arrival time**

Xu Cui, Chess Stetson, Read Montague, David Eagleman

In this supplementary material, we provide further details about our methods and address some additional points of analysis.

***Can the amplitude of the fMRI signal be explained by reaction time only? No.***

It has been previously documented that reaction times are related to the preceding readiness period—this is known as the variable foreperiod effect.^1-3^ The fMRI amplitude reported by us is also related to the readiness period (see main text) – might this suggest the fMRI amplitude is simply explained by reaction time? We show here that while they may be subserved by common circuitry, neither phenomenon can directly explain the other. First, the go/no-go task demonstrates that no reaction is necessary for the fMRI effect. Secondly, note by way of example that the reaction times for the 8, 10 and 12 sec readiness periods is almost the same (Figure S1), even while the corresponding fMRI signal is still dependent on the readiness period (Figure 1c,d). This indicates that the reaction time effects are separable from the fMRI signal effects reported here. To solidify this conclusion, we performed a linear regression in which we orthogonalized reaction time and readiness period. Specifically, the residuals from using reaction time as a regressor were highly correlated with readiness period in the SMA (*p*=1.8x10^-6^) and in STG (*p*=3.3x10^-7^).

***Is the post-go fMRI signal detectable in previous studies? Perhaps.***

A recent neuroimaging report by Curtis and Connolly (2008) appears to have found quite different results than ours. However, an analysis of their transverse parietal sulcus time course, recentered at the time of the go cue, shows what appears to be post-go fMRI activity very similar to ours (Figure S2). Note that since this is found in a different brain region, and saccades were used instead of button presses, more investigation is warranted before we can conclude this is the same effect.

**References**

1. Niemi, P. & Naatanen, R. Foreperiod and Simple Reaction-Time. *Psychol. Bull.* **89**, 133-162 (1981).

2. Churchland, M. M., Yu, B. M., Ryu, S. I., Santhanam, G. & Shenoy, K. V. Neural variability in premotor cortex provides a signature of motor preparation. *J. Neurosci.* **26**, 3697-3712 (2006).

3. Pellizzer, G., Hedges, J. H. & Villanueva, R. R. Time-dependent effects of discrete spatial cues on the planning of directed movements. *Exp. Brain Res.* **172**, 22-34 (2006).

4. Curtis, C.E. and J.D. Connolly, Saccade preparation signals in the human frontal and parietal cortices. *J Neurophysiol*, 2008. 99(1): p. 133-45.
